# Ureterosciatic Hernia in Focus: A Narrative Review of the Literature

**DOI:** 10.7759/cureus.49895

**Published:** 2023-12-04

**Authors:** Mohamed Mustafa, Afiq Pouzi, Peter Senada, Lokesh Suraparaju, Suresh Gupta

**Affiliations:** 1 Urology, James Paget University Hospitals NHS Foundation Trust, Great Yarmouth, GBR

**Keywords:** obstructive uropathy, sciatic ureteral hernia, curlicue sign, lindbom hernia, uretero-sciatic hernia

## Abstract

Pelvic herniation of the ureter through anatomical musculoskeletal foramina stands out as one of the rarest causes of ureteric obstruction. Historically, most cases have been documented as incidental intraoperative findings. The herniation of the ureter through the sciatic foramen presents as a particularly uncommon variant of this condition, distinguished by its potential to cause life-threatening sepsis or renal failure if not promptly recognized and treated. The diagnostic process remains challenging, attributed partly to the vague initial symptomatology and subtle radiological findings, and second, to the rarity of this condition. This challenge may be further compounded by the lack of a clear description of clinical features and pathways to raise clinician suspicion. In light of these considerations, we conducted this literature review to illuminate this unique cause of obstructive uropathy, aiming to delineate its clinical features and explore common diagnostic and treatment options.

## Introduction and background

**Figure 1:**
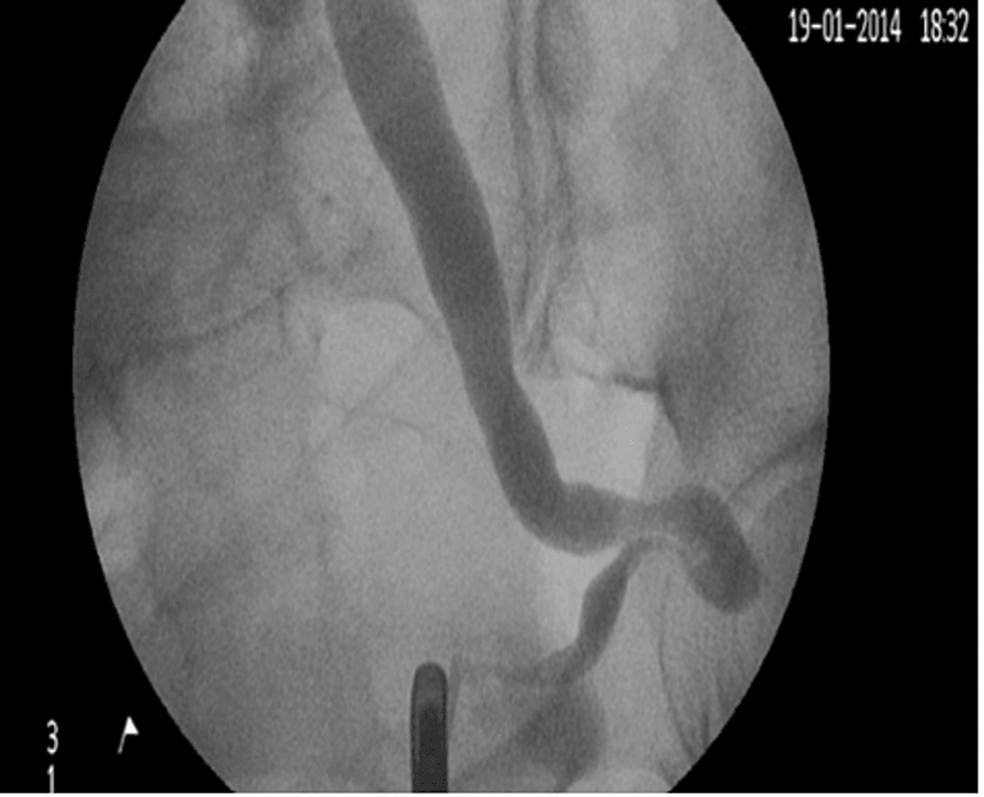
Retrograde pyelogram demonstrates distal ureter curlicue-like obstruction. This figure is obtained from the author’s previous publication [4].

**Figure 2:**
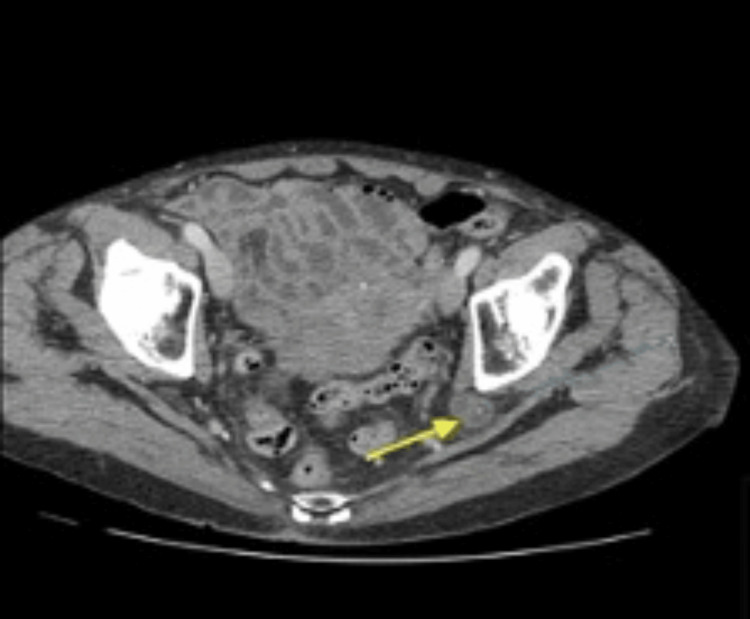
CT scan axial section demonstrates a dilated ureter outside the ureter. This figure is obtained from the author’s previous publication [4].

Herniation of the ureters through the sciatic foramen is a notably uncommon occurrence, ranking third in frequency after inguinal and femoral hernias [1]. However, within reported cases spanning from 1900 to 2008, ureter-containing sciatic hernias emerged as the second most frequently documented type [2]. The inception of awareness in this field can be traced back to the pioneering work of Swedish radiologist Ake Lindbom in 1947, who identified and reported the first case of uretrosciatic hernia, subsequently labeled the Lindbom hernia [3]. Lindbom contributed significantly to the understanding of this hernia by delineating its classical radiological sign, now recognized as the “curlicue sign.” This distinctive sign is observable through diagnostic modalities such as retrograde pyelogram or computed romography (CT) (refer to Figure 1 and Figure 2, respectively) [4].

Anatomically, the sciatic notch is divided into lesser and greater foramina by the sacrotuberous and sacrospinous ligaments. The greater sciatic foramen is further partitioned by the piriformis muscle into supra-piriformis and infra-piriformis compartments. Sciatic hernias typically manifest through the supra-piriformis compartment on either side [1]. However, occurrences through the lesser sciatic foramen have also been documented [2].

The pathogenesis of sciatic herniation is not clearly understood, likely due to its rarity. Speculations suggest that concomitant atrophy of the piriformis muscle, coupled with an increase in intra-abdominal pressure, may be predisposing factors. Piriformis muscle atrophy can result from neuromuscular disease, hip disease, or other locomotor disturbances [3]. Additionally, it has been proposed that ureteral herniation occurs through a parietal pelvic fascia defect at the site of penetration of the superior or inferior gluteal or pudendal arteries [5]. In the younger population, occurrences are evenly distributed between genders, while in adults, females seem to be more commonly affected [3].

Clinically, patients’ symptoms at presentation can mimic renal colic, characterized by pain from the loin to the groin, or may manifest with sepsis and/or septic shock. Radiological imaging plays a crucial role in both establishing the diagnosis and identifying alternative causes of ureteral obstruction. The presence of the “curlicue sign” in imaging is pathognomonic for ureterosciatic hernia, providing a distinctive marker for accurate diagnosis [6]. Management options have evolved over the past five decades, mirroring advancements in the field since the first case reported by Lindbom [7].

Managing these patients can pose a challenge initially, as the diagnosis may not be immediately apparent. However, the fundamental principles of managing an acutely ill surgical patient remain consistent, focusing on resuscitation and stabilization. Definitive management presents a point of contention, with some studies advocating for immediate surgical repair, whether laparoscopic, robotic-assisted, or open, while others opt for initial patient stabilization through a temporizing stent, followed by subsequent definitive surgical repair or long-term maintenance with stent changes.

In this article, we present a narrative review encompassing all published cases in the English-language literature. Our objective is to draw conclusions or propose a plausible pattern of clinical features associated with this condition. Additionally, we aim to provide a comprehensive review of management options and their respective outcomes.

## Review

Methodology

A comprehensive literature search was conducted, encompassing databases such as PubMed and Scopus. Search terms and keywords, including “Ureterosciatic hernia,” “Lindbom hernia,” “ureteric sciatic hernia,” and “ureteral obstruction,” were employed. The results were meticulously screened based on titles and abstracts, with the exclusion of non-English papers, review articles, and letters to the editor. After this initial screening, repeated articles across databases were identified and eliminated. The final step involved creating a comprehensive spreadsheet compiling all published case reports. This spreadsheet served as a valuable tool for facilitating the subsequent analysis and summarization of clinical features, management options, and outcomes.

Results

An initial database search yielded 70 papers dating back to 1947. Following the screening of titles and abstracts, 17 articles were excluded. Subsequent full-text screening of the remaining 53 articles resulted in a final list of 47 case reports. These reports have been meticulously included in this review and are summarized in Table 1 below.

**Table 1:** Summary of published reports.

Case number	Authors	Year	Age (years)	Gender	Side affected	Presentation	Imaging	Initial management	Definitive management	Complications
1	Lindbom [6]	1947	54	Female	Left supra-piriformis	Left flank pain and sepsis	Retrograde pyelography	Retrograde ureteric stenting	Resection and reimplantation of the ureter	None
2	Beck et al. [5]	1952	66	Female	Left supra-piriformis	Intermittent left flank pain and sepsis	IV urogram and retrograde pyelography	Not reported	Surgical reduction of hernia and lateral fixation of the ureter	None
3	Franken and Smith [7]	1969	58	Female	Bilateral infra-piriformis	Intermittent upper abdominal pain	IV urogram	Not reported	Laparotomy and open hernia repair	None
4	Rothchild [8]	1969	65	Female	Left infra-piriformis	Intermittent left flank pain	Retrograde pyelogram	Not reported	Laparotomy and open hernia repair	None
5	Spring et al. [9]	1983	65	Male	Right infra-piriformis	Incidental finding	CT urogram and excretory urogram	Not reported	Not reported	None
6	Oyen et al. [10]	1987	75	Female	Left infra-piriformis	Left flank pain and sepsis	CT AP	Conservative	Conservative	None
7	Stöckle et al. [11]	1989	65	Female	Left supra-piriformis	Left flank pain with sepsis	Antegrade and retrograde pyelogram	Nephrostomy and manual reduction by pressure to the left gluteal area	Open hernia repair	None
8	Rommel et al. [4]	1993	64	Male	Left supra-piriformis	Chronic left flank pain	IV urogram and retrograde pyelography	Conservative	Surgical reduction of hernia and fixation of the ureter	None
9	Epner et al. [12]	1994	86	Female	Left supra-piriformis	Recurrent pyuria without flank pain	IV urogram	Conservative	Conservative	None
10	Arat et al. [13]	1996	66	Female	Greater sciatic foramen	Not disclosed	Spiral CT scan	Conservative	Conservative	None
11	Ritschel et al. [14]	1996	51	Female	Left supra-piriformis	Left flank pain	CT AP	Nephrostomy and trial of retrograde ureteric stenting	Surgical reduction and hernia repair	None
12	Gee et al [15]	1999	60	Female	Left sciatic foramen	Left flank pain	Retrograde pyelography	Retrograde ureteric stenting	Laparoscopic hernia mesh repair	None
13	Weintraub et al. [3]	2000	87	Female	Right sciatic foramen	Incidental finding	CT AP	Percutaneous nephrostomy	Antegrade stenting	None
14	Touloupidis et al. [16]	2006	61	Female	Right infra-piriformis	Symptoms of sciatic nerve compression by the hernia	IV urogram and retrograde ureterography	Conservative	Ureterolysis, reimplantation of the ureter, and hernia mesh repair	None
15	Loffroy et al. [17]	2007	81	Female	Left sciatic foramen	Sepsis	Antegrade pyelogram	Percutaneous nephrostomy	Ureteric resection and anastomosis with hernia repair	None
16	Witney-Smith et al. [18]	2007	59	Female	Left sciatic foramen	Sepsis	CT urogram	Retrograde ureteric stenting	Laparoscopic repair of hernia and ureteric stent	None
17	Tsai et al. [19]	2008	91	Female	Left sciatic foramen	Incidental finding	CT AP	Conservative	Watchful waiting	None
18	Clemens et al. [20]	2010	80	Female	Left sciatic foramen	Left flank pain	CT AP	Retrograde ureteric stenting	Routine ureteric stent changes	None
19	Hsu et al. [21]	2010	69	Female	Left sciatic foramen	Left flank pain	CT AP	Retrograde ureteric stenting	Stented for three months	None
20	Sugimoto et al [22]	2011	76	Female	Left sciatic foramen	Left flank pain	CT KUB	Retrograde ureteric stenting	Stented for two months	None
21	Singh et al. [23]	2013	75	Female	Left sciatic foramen	Left flank pain and sepsis	CT AP and retrograde pyelography	Percutaneous nephrostomy and antegrade ureteric stenting	Robot-assisted laparoscopic hernia repair	None
22	Whyburn et al. [24]	2013	74	Female	Bilateral ureteric hernia	Bilateral flank pain and renal failure	CT AP	Retrograde ureteric stent	Bilateral ureterolysis and hernia mesh repair	None
23	Kato et al. [25]	2014	72	Female	Left sciatic foramen	Left flank pain	IV urogram	Retrograde ureteric stenting	Stented for three months	None
24	Tsuzaka et al. [26]	2014	78	Female	Left sciatic foramen	Left flank pain	CT urogram	Conservative	Laparoscopic hernia repair	None
25	Salari et al. [27]	2015	87	Female	Right sciatic foramen	Right flank pain	Retrograde pyelogram	Retrograde ureteric stenting	Routine ureteric stent changes	None
26	Yanagi et al. [28]	2015	92	Female	Left sciatic foramen	Fever and vomiting	CT AP and retrograde pyelography	Retrograde ureteric stenting	Routine ureteric stent changes	None
27	Regelman et al. [29]	2016	60	Female	Left supra-piriformis	Left flank pain	CT urogram	Conservative	Robotic-assisted hernia repair	None
29	Wai et al. [30]	2016	68	Female	Left sciatic foramen	Left flank pain	CT urogram	Percutaneous nephrostomy	Laparotomy and open reduction and hernia mesh repair	Rupture of the renal pelvis
30	Demetriou et al. [31]	2016	76	Female	Left sciatic foramen	Right flank pain	CT urogram	Conservative	Conservative	None
31	Nakazawa et al. [32]	2018	92	Female	Left sciatic foramen	Left flank pain	CT AP	Retrograde ureteric stenting	Stented for two months	None
32	Destan et al. [33]	2019	80	Female	Right sciatic foramen	Right flank pain	CT AP	Retrograde ureteric stenting	Robotic-assisted ureteric resection and anastomosis	None
33	Kimura et al. [34]	2019	86	Female	Left sciatic foramen	Left flank pain	CT KUB	Ultrasound-guided manual transvaginal reduction	Conservative	None
34	Moon et al. [35]	2019	72	Female	Right sciatic foramen	Right flank pain	CT AP	Percutaneous nephrostomy	Laparoscopic reduction and hernia repair	None
35	Kamisawa et al. [36]	2020	70	Female	Right sciatic foramen	Right abdominal pain	CT AP	Conservative	Laparoscopic intra-peritonization	None
36	Rose et al. [37]	2020	68	Female	Left sciatic Foramen	Left flank pain	CT urogram	Percutaneous nephrostomy	Robot-assisted hernia repair	None
37	Kim et al. [38]	2020	68	Female	Left sciatic foramen	Left flank pain	CT AP and pyelography	Conservative	Ureteric balloon dilatation and stenting	None
38	Kubota et al. [39]	2020	85	Female	Left sciatic foramen	Septic shock	CT AP and pyelography	Conservative	Laparoscopic hernia mesh repair	None
39	Nagasubramanian et al. [40]	2020	57	Female	Left sciatic foramen	Left flank pain	CT scan and pyelography	Retrograde ureteric stenting	Laparoscopic hernia reduction and repair	None
40	Sechrist et al. [41]	2021	75	Female	Right sciatic foramen	Asymptomatic	CT AP	Conservative	Conservative	None
41	Chan et al. [42]	2021	97	Female	Left sciatic foramen	Sepsis	CT AP with contrast	Retrograde ureteric stenting	Routine ureteric stenting	None
42	Kakimoto et al [43]	2021	86	Female	Left sciatic foramen	Septic shock	CT AP with contrast	Retrograde ureteric stenting	Conservative	Mortality within 32 days
43	Li et al [44]	2022	72	Female	Right sciatic foramen	Intermittent right flank pain and sepsis	CT scan and pyelography	Conservative	Laparoscopic reduction of ureter and hernia repair	None
44	Mustafa et al [45]	2022	52	Female	Left sciatic foramen	Left flank pain and sepsis	CT AP and IV urogram	Retrograde ureteric stenting	Routine stenting	None
45	Shibata et al. [46]	2023	90	Female	Left sciatic Foramen	Left gluteal pain and swelling	CT AP and retrograde pyelography	Retrograde ureteric stenting	Routine stenting	Gluteal abscess
46	Yanagida et al. [47]	2023	83	Female	Left sciatic Foramen	Left flank pain and sepsis	CT AP	Retrograde ureteric stenting	Stented for eight months	None
47	Fridling e al. [48]	2023	73	Female	Left sciatic foramen	Left flank pain	CT AP	Retrograde ureteric stenting	Robotic-assisted hernia mesh repair	None

Cases in this review spanned from 1947 to 2023, with all patients being above the age of 50 (ranging from 51 to 97 years old), and only two cases reported among male patients. Radiological diagnosis, whether through CT scans or pyelography, was consistent across all patients.

Among the reported cases, 10 had a right-sided hernia, 34 exhibited a left-sided ureterosciatic hernia, and two presented with bilateral herniation of the ureters into the sciatic foramina. The most frequent symptom at presentation was flank pain, noted in 26 cases, while 16 cases presented with pain accompanied by signs of active infection, sepsis, and septic shock. Additionally, five cases were incidentally diagnosed during imaging for unrelated reasons.

In terms of management, the majority of cases underwent initial temporizing emergency procedures, followed by definitive surgical interventions. Specifically, 19 cases received a temporizing stent, eight cases were initially managed with percutaneous nephrostomy, and 18 cases had no initial surgical intervention. The primary definitive management approach involved a surgical reduction of the ureter with hernia site repair, performed in 23 cases using open, laparoscopic, or robotic-assisted methods. The second most common treatment approach was ureteric stenting, implemented in 13 cases for varying durations ranging from two to eight months. Notably, only one mortality was reported, attributed to a presentation with septic shock and an emergency retrograde stenting.

Discussion

Clinical Features

The predominant clinical feature in these cases was the description of flank pain, which could be either unilateral or bilateral. This pain presentation varied, with or without systemic signs indicative of superimposing infection. The inherent reducibility of hernias, a common characteristic, might elucidate reports of intermittent flank pain or chronic side pain. Additionally, some cases were incidentally discovered during imaging for unrelated reasons. Furthermore, reports indicate additional clinical features, including symptoms and episodes of recurrent urinary tract infections [13], pressure symptoms from the hernia in the form of gluteal pain, or features of sciatic nerve compression [17,46].

Radiological Investigations

Urological imaging has seen limited advancements over the years, which is reflected in the diagnostic modalities for this condition. Nevertheless, the demonstration of the “curlicue sign or ureter” has consistently been considered pathognomonic for ureterosciatic hernia, first reported and described by Lindbom [7]. The term “curlicue ureter” was later coined in the second reported case, a few years afterward, in a paper by Beck et al. [6].

Initially, retrograde pyelography and intravenous urography (IVU) were employed to showcase the presence of a loop of the ureter passing through the sciatic foramen. Historically, IVU demonstrated a sensitivity of 66-87% and a specificity of 92-94% [49,50]. However, with the advent of CT scanners, their increased availability, and the superior information they provide compared to IVUs, the practice has shifted more toward utilizing CT scans as the primary imaging modality. The introduction of contrast medium enhances accuracy and provides a better anatomical demonstration of the herniated ureter through the sciatic foramen, aiding in surgical planning.

Some cases have reported an additional step of intraoperative pyelogram, typically performed at the time of ureteric stent placement, to enhance accuracy and confirm the diagnosis. However, clinicians need to be aware that the hernia might reduce spontaneously, leading to different imaging findings.

Management and Treatments

Generally, management options for pelvic herniation of the ureter through sciatic foramina encompass observation (primarily for asymptomatic patients), ureteral stenting, and surgical correction. However, for septic and acutely unwell patients facing significant risks associated with general anesthesia and surgical procedures, decompressing the system using nephrostomies has been reported.

Decompression with a double JJ stent is generally feasible without requiring special skills or expertise. It offers the advantage of allowing time for definitive surgical planning while maintaining a patent system, thus reducing the risks of recurrence or the development of fulminant sepsis. Notably, some cases have been managed solely with routine long-term ureteric stent changes, spanning from a few years to several decades [4,21,46].

On the other hand, there were reports of brief periods with a double JJ stent as the primary mode of management, ranging between two and eight months [22,23,26,33,47]. These findings suggest the feasibility of employing double-J stents as the main modality for management. However, there is currently no clear evidence indicating an optimum period for using stents. In the authors’ view, the main drawback of long-term stenting is the necessity for repeated procedures, carrying potential risks and side effects. Additionally, there is a risk of stent failure to reduce the hernia [19].

The initial approach for definitive management involved the resection of the herniating ureteric segment and re-implantation of the ureter [7,18]. Other studies have reported an open reduction of the herniated segment of the ureter without the need for resection, accompanied by the repair of the defect in the sciatic foramen [8,9,12,15,19,31], modifications to this open approach included the fixation of the ureter to the posterior abdominal wall [6]. The open surgical repair of Lindbom hernia persisted as the standard approach for several years, even with the advancements in minimally invasive surgical technologies. It took 52 years since the first case report by Lindbom for the first paper describing the laparoscopic approach to repair this hernia to be published in 1999 [16]. Since then, other cases have reported similar laparoscopic approaches with favorable results and no reports of significant complications or recurrences. The reported techniques consistently involved a three or four-port approach in a semi-lateral position. The procedure typically includes the release of the herniated segment of the ureter, followed by the obliteration of the hernia defect, either anatomically or through the use of an appropriate mesh.

## Conclusions

Ureterosciatic hernia poses a diagnostic challenge, emphasizing the importance of clinician awareness regarding ureteral hernias as a potential cause of obstructive uropathy, particularly in the elderly female population. Radiological confirmation is crucial for an accurate diagnosis. While various surgical techniques have been described in the literature, the majority of cases have shown a satisfactory response to ureteric stenting. Long-term stent placements can be considered definitive management for many cases. With increasing accessibility and technological developments in radiology, we expect more similar cases to be reported and management strategies to evolve more. We believe future research can look into comparing the outcomes of surgical approaches and establish a consensus on the optimum approach.

## References

[1] Lebowitz RL (1973). Ureteral sciatic hernia. Pediatr Radiol.

[2] Pollack HM, Popky GL, Blumberg ML (1975). Hernias of the ureter.--an anatomic-roentgenographic study. Radiology.

[3] Weintraub JL, Pappas GM, Romano WJ, Kirsch MJ, Spencer W (2000). Percutaneous reduction of ureterosciatic hernia. AJR Am J Roentgenol.

[4] Rommel FM, Boline GB, Huffnagle HW (1993). Ureterosciatic hernia: an anatomical radiographic correlation. J Urol.

[5] Beck WC, Baurys W, Brochu J, Morton WA (1952). Herniation of the ureter into the sciatic foramen ("curlicue ureter"). J Am Med Assoc.

[6] Lindbom A (1947). Unusual ureteral obstruction by herniation of ureter into sciatic foramen. Acta radiol.

[7] Franken EA Jr, Smith EE (1969). Sciatic hernia: report of three cases including two with bilateral ureteral involvement. Am J Roentgenol Radium Ther Nucl Med.

[8] Rothchild TP (1969). Ureteral hernia. Report of a case of herniation of the ureter into the sciatic foramen. Arch Surg.

[9] Spring DB, Vandeman F, Watson RA (1983). Computed tomographic demonstration of ureterosciatic hernia. AJR Am J Roentgenol.

[10] Oyen R, Gielen J, Baert L, Van Poppel H, Baert AL (1987). CT demonstration of a ureterosciatic hernia. Urol Radiol.

[11] Stöckle M, Müller SC, Riedmiller H (1989). Ureterosciatic hernia. A rare cause of pyonephrosis. Eur Urol.

[12] Epner SL, Lautin EM (1994). Case report: intermittent sciatic herniation of the ureter. Clin Radiol.

[13] Arat A, Haliloglu M, Cila A, Demirkazik F, Balkanci F (1996). Demonstration of ureterosciatic hernia with spiral CT. J Comput Assist Tomogr.

[14] Ritschel S, Heimbach D, Schoeneich G (1996). Ureterosciatic hernia. Scand J Urol Nephrol.

[15] Gee J, Munson JL, Smith JJ 3rd (1999). Laparoscopic repair of ureterosciatic hernia. Urology.

[16] Touloupidis S, Kalaitzis C, Schneider A, Patris E, Kolias A (2006). Ureterosciatic hernia with compression of the sciatic nerve. Int Urol Nephrol.

[17] Loffroy R, Bry J, Guiu B, Dubruille T, Michel F, Cercueil JP, Krausé D (2007). Ureterosciatic hernia: a rare cause of ureteral obstruction visualized by multislice helical computed tomography. Urology.

[18] Witney-Smith C, Undre S, Salter V, Al-Akraa M (2007). An unusual case of a ureteric hernia into the sciatic foramen causing urinary sepsis: successfully treated laparoscopically. Ann R Coll Surg Engl.

[19] Tsai PJ, Lin JT, Wu TT, Tsai CC (2008). Ureterosciatic hernia causes obstructive uropathy. J Chin Med Assoc.

[20] Clemens AJ, Thiel DD, Broderick GA (2010). Ureterosciatic hernia. J Urol.

[21] Hsu HL, Huang KH, Chang CC, Liu KL (2010). Hydronephrosis caused by ureterosciatic herniation. Urology.

[22] Sugimoto M, Iwai H, Kobayashi T, Morokuma F, Kanou T, Tokuda N (2011). Ureterosciatic hernia successfully treated by ureteral stent placement. Int J Urol.

[23] Singh I, Patel B, Hemal AK (2013). Robotic repair of a rare case of symptomatic "ureterosciatic hernia". Indian J Urol.

[24] Whyburn JJ, Alizadeh A (2013). Acute renal failure caused by bilateral ureteral herniation through the sciatic foramen. Urology.

[25] Kato T, Komiya A, Ikeda R, Nakamura T, Akakura K (2015). Minimally invasive endourological techniques may provide a novel method for relieving urinary obstruction due to ureterosciatic herniation. Case Rep Nephrol Dial.

[26] Tsuzaka Y, Saisu K, Tsuru N, Homma Y, Ihara H (2014). Laparoscopic repair of a ureteric sciatic hernia: report of a case. Case Rep Urol.

[27] Salari K, Yura EM, Harisinghani M, Eisner BH (2015). Evaluation and treatment of a ureterosciatic hernia causing hydronephrosis and renal colic. J Endourol Case Rep.

[28] Yanagi K, Kan A, Sejima T, Takenaka A (2015). Treatment of ureterosciatic hernia with a ureteral stent. Case Rep Nephrol Dial.

[29] Regelman M, Raman JD (2016). Robotic assisted laparoscopic repair of a symptomatic ureterosciatic hernia. Can J Urol.

[30] Wai OK, Ng LF, Yu PS (2016). Ruptured renal pelvis due to obstruction by ureterosciatic hernia: a rare condition with a rare complication. Urology.

[31] Demetriou GA, Perera S, Halkias C, Ahmed S (2016). Seventy-six-year-old woman with an unusual anatomy of the left ureter. BMJ Case Rep.

[32] Nakazawa Y, Morita N, Chikazawa I, Miyazawa K (2018). Ureterosciatic hernia treated with ureteral stent placement. BMJ Case Rep.

[33] Destan C, Durand X (2019). Management of Lindbom's hernia (ureterosciatic hernia). J Visc Surg.

[34] Kimura J, Yoshikawa K, Sakamoto T, Lefor AK, Kubota T (2019). Successful manual reduction for ureterosciatic hernia: a case report. Int J Surg Case Rep.

[35] Moon KT, Cho HJ, Choi JD, Kang JY, Yoo TK, Cho JM (2019). Laparoscopic repair of a ureterosciatic hernia with urosepsis. Urol J.

[36] Kamisawa K, Ohigashi T, Omura M, Takamatsu K, Matsui Z (2020). Ureterosciatic hernia treated with laparoscopic intraperitonization of the ureter. J Endourol Case Rep.

[37] Rose KM, Carras K, Arora K, Pearson D, Harold K, Tyson M (2020). Robot-assisted repair of ureterosciatic hernia with mesh. J Robot Surg.

[38] Kim YU, Cho JH, Song PH (2020). Ureterosciatic hernia causing obstructive uropathy successfully managed with minimally invasive procedures. Yeungnam Univ J Med.

[39] Kubota M, Makita N, Inoue K, Kawakita M (2020). Laparoscopic repair of ureteral diverticulum caused by ureterosciatic hernia. Urology.

[40] Nagasubramanian S, George AJ, Chandrasingh J (2020). Case of ureterosciatic hernia managed by laparoscopic repair. ANZ J Surg.

[41] Sechrist E, Elmaoued A, Ong CJ, Trivedi S, Gerena M, Wagner R, Allam E (2021). Ureterosciatic hernia with concomitant Amyand hernia: case report and review of the literature. Radiol Case Rep.

[42] Chan CY, Lai TC, Yu CH, Leung CL, Chan WK, Law IC (2021). Ureterosciatic hernia with pyonephrosis and obstructive uropathy: a case report. Hong Kong Med J.

[43] Kakimoto K, Hikone M, Nagai K, Yamakawa J, Sugiyama K, Hamabe Y (2021). Urosepsis secondary to ureterosciatic hernia corrected with ureteral stent placement: a case report and literature review. Int J Emerg Med.

[44] Li B, Wang Y, Sun Y, Man Y, Zhang X (2022). One case report of laparoscopic biological patch repair for the ureterosciatic hernia and literature review. Heliyon.

[45] Mustafa M, Suraparaju L, Lyons M, Gupta S (2022). Uretero-sciatic hernia (Lindbom hernia): a case report and long-term follow-up. Cureus.

[46] Shibata Y, Ueda T (2023). Ureterosciatic hernia with gluteal abscess: a case report. Urol Case Rep.

[47] Yanagida K, Watanabe D, Yoshida T, Kawae N, Mizushima A, Nakagawa T (2023). Obstructive urosepsis caused by ureterosciatic hernia: a case report. Urol Case Rep.

[48] Fridling J, Gontarz B, Stein J, Daoud V (2023). Robotic-assisted repair of a ureterosciatic hernia with combined ureteral stenting. CRSLS.

[49] Imran F, Zaman Z, Iqbal MJ (2017). Diagnostic accuracy of IVU compared to unenhanced CT KUB for detection of urinary tract calculi. J Islamabad Med Dent Coll.

[50] Pfister SA, Deckart A, Laschke S (2003). Unenhanced helical computed tomography vs intravenous urography in patients with acute flank pain: accuracy and economic impact in a randomized prospective trial. Eur Radiol.

